# Association of Site and Recurrence in Oral Squamous Cell Carcinoma Patients Visiting Private Hospital in Chennai: A Retrospective Study

**DOI:** 10.7759/cureus.52774

**Published:** 2024-01-23

**Authors:** Prathiba Reichal, Pratibha Ramani, Suvarna Kizhakkoottu

**Affiliations:** 1 Dentistry, Saveetha Dental College and Hospitals, Saveetha Institute of Medical and Technical Sciences, Saveetha University, Chennai, IND; 2 Oral Pathology and Microbiology, Saveetha Dental College and Hospitals, Saveetha Institute of Medical and Technical Sciences, Saveetha University, Chennai, IND

**Keywords:** prevalence, lesional site, recurrence, oscc, oral squamous cell carcinoma, oral cancer, head and neck cancer

## Abstract

Background: Oral squamous cell carcinoma (OSCC) is the most common malignant neoplasms of the oral cavity. Tongue, buccal mucosa, and gingivobuccal sulcus are the most commonly involved sites for the local recurrence of OSCC. The site of the tumor can be a critical parameter in relation to the recurrence of OSCC because of the varied action of tumor cells in different tumor macro and microenvironments. Hence, the current study aims to evaluate the correlation between the site and recurrence of OSCC among patients visiting private oral cancer hospitals.

Materials and methods: Details of n=300 OSCC cases reported during 2019-2023, which included primary and recurrent OSCC, were collected. The sample population selected includes 261 primary and 39 recurrent oral squamous cell carcinoma cases. The demographic and clinicopathological data were retracted from the institution's common clinical database and transported to IBM-SPSS 23 software for statistical analysis. Chi-square was done to evaluate the association between site and recurrence status, and p<0.05 was considered statistically significant.

Results: Males have a high predilection for OSCC, and the recurrent cases account for 13% of the sample population. The buccal mucosa was the most commonly affected site in primary and recurrent OSCC cases. However, the association between the site of the lesion and the status of recurrence was found to be statistically significant, with a p-value of 0.001.

Conclusion: Even though buccal mucosa was the most common site for recurrent OSCC(p value-0.001), the present study carries a small sample size and a location-specific sampling. Hence, further studies must be conducted with a large sample size to test the significant correlation between the site and recurrence rate among patients diagnosed with OSCC.

## Introduction

Oral squamous cell carcinoma (OSCC) constitutes the most common type of malignancy among Head and neck neoplasms. According to the Global Cancer Observatory in 2020, the annual incidence of OSCC was 377,713 cases worldwide, with the highest number recorded in Asia (248,360), followed by Europe (65,279) and North America (27,469) [1]. In India, the incidence of oral cancer is higher, the cases are reported at an advanced stage, and the chances of cure are less, leaving five-year survival rates around 20% only [2].

In India, the commonly established risk factors of OSCC are tobacco and alcohol. Tobacco smoking has a prevalence of causing oral cancer in about 75% of individuals [3,4]. Tobacco smoking has a six-fold risk of causing oral squamous cell carcinoma when compared with the non-smoking population [5,6]. Similarly, a six-fold increase in the development of OSCC is seen in alcoholics compared with non-drinkers. There is a fifteen times increase in the causation of oral cancer when there is a combined action of tobacco smoking and alcohol [7,8]. Betel quid chewing and the use of areca nut are predominant causative agents of oral cancer in India and the Taiwanese population. On par with them, narcotics and cannabis also cause oral cancer [9]. 

OSCC represents a specific pathological type of cancer occurring in the oral cavity and oropharynx. However, distinguishing between the site of the lesion is important because the tissue surrounding the tumor can act as a channel for tumor invasion directly into the underlying structures and to the regional or distant lymph nodes as metastatic deposits. Oral cancer that develops at various anatomic subsites may become more advanced, depending on the site of involvement, reiterating the importance of anatomical sites [10,11]. A study by Nair et al. 2016 reported significant differences in survival, clinical, and pathological features between tongue and buccal OSCC [12]. Few other studies also suggested the variation in the clinicopathological behavior of OSCC in accordance with the site of occurrence [13-15]. However, these studies could not investigate OSCC arising from rare anatomic sites such as the floor of the mouth since the availability of such cases was scarce. 

In the case of recurrent OSCC, the site of recurrence is also important. The tongue, buccal mucosa, and gingivobuccal sulcus are the most commonly involved sites for the local recurrence of OSCC [16]. Certain researchers have validated that pathological variables like TNM stage, depth of invasion, surgical margin status, and pattern of invasion could be reliable parameters for predicting the probability of recurrence [17]. The site of the tumor may be critical for local control. Previous studies by Deshmukh et al. and Suresh et al. stated that the tumors on the oral floor and buccal mucosa had worse local control than other sites within the oral cavity [18]. Hence, the current study aimed to determine whether there is any association between the site and recurrence of oral squamous cell carcinoma patients visiting a private oral cancer hospital during a specific period.

## Materials and methods

The present study was conducted to analyze the association between site and recurrence in OSCC cases reported to Saveetha Dental College and Hospitals, Chennai. This study was carried out after getting approval from the Institutional ethical committee with ethical number SDC/SIHEC/2020/DIASDATA/0619-0320. The present study entailed a total sample size of n=300, which included 261 primary OSCC cases and 39 recurrent OSCC cases reported to the Department of Oral Pathology and Microbiology during the period of three years(2019-2022). We Independently reviewed the histopathology reports, history of surgical management, and review reports for all the cases reported from 2019-2022. We segregated the reported cases into two groups, recurrent OSCC(70) and primary OSCC(1000). On further review of the histopathology reports of the segregated cases, cases with less than or equal to 1 year of duration between primary and recurrence and also cases with involved surgical margins were excluded from the recurrent OSCC group, resulting in a final recurrent OSCC sample size of n=39. We adhered to this exclusion criteria in the sampling of recurrent OSCC in order to limit the bias from the inadequate surgical removal of the tumor during surgical excision. Initially, segregated primary OSCC cases were further evaluated based on their histopathological reports. The sampling method used in the primary OSCC group was random sampling. This method was used to limit the recurrent primary ratio to 1:6; thus, the final sample size was brought down to n=261. The demographic and clinicopathological details were retracted from the institution's common clinical database. The demographic details of selected cases, such as age and gender, and clinicopathological details, such as site of lesion and status of recurrence, were extracted and segregated into Microsoft Excel sheets. The collected data were reviewed from 2019 to 2022 by 2 independent reviewers to check the inclusion criteria and reduce discrepancy and repetition of cases. The segregated data were then transferred to IBM-SPSS 23 software for statistical analysis. Descriptive analysis tools were used to evaluate the prevalence of gender, recurrence, and site of the lesion. Pearson Chi-Square test is used to analyze whether there is an association between recurrence and the site of the lesion. In the Pearson Chi-Square test, p-value <0.05 was considered statistically significant.

## Results

**Figure 1:**
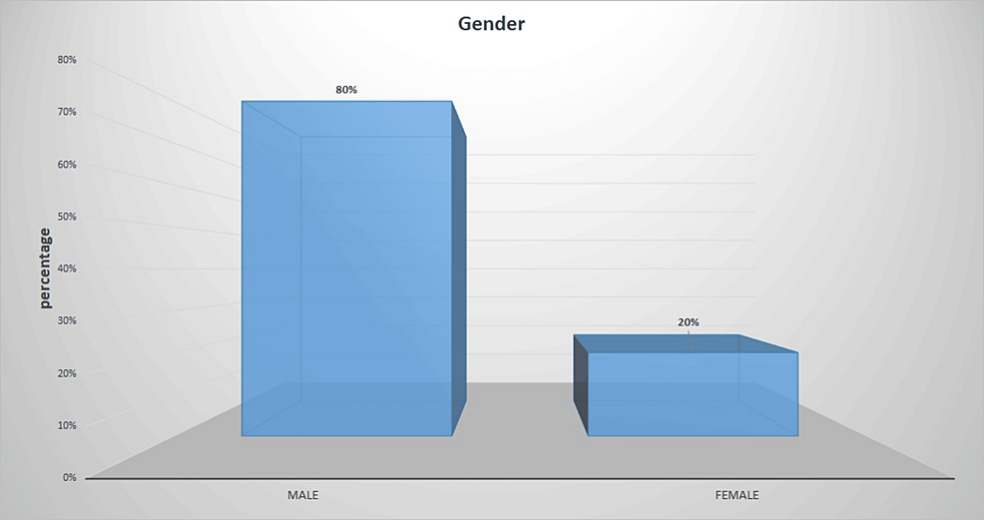
Shows the gender distribution of the selected sample population of OSCC cases. The X-axis represents the gender, while the Y-axis represents the percentage of cases. 80% of the sample population were male and 20% were females. OSCC: Oral squamous cell carcinoma

**Figure 2:**
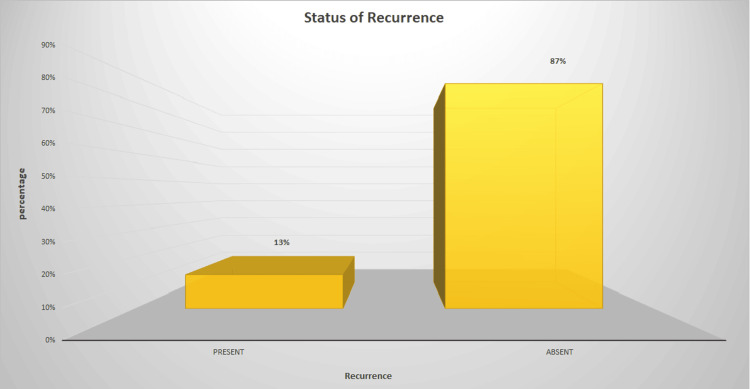
Depicts the percentage of recurrent and primary OSCC cases among the selected sample population. The X-axis represents the status of recurrence, and the Y-axis represents the percentage of cases OSCC: Oral squamous cell carcinoma

Among 300 OSCC cases studied, 20% (60) of the study population were females, and 80% (240) were males (Figure 1). The majority of the selected cases (87%) (261) were primary OSCC; however, 13% (39) of cases were recurrent OSCC (Figure 2).

**Figure 3:**
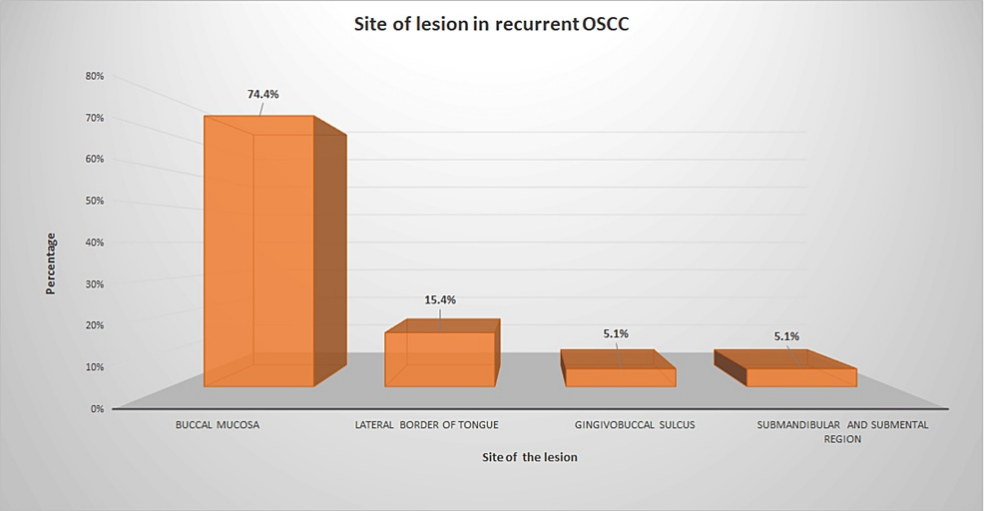
Depicts the percentage of recurrent OSCC cases among the sample population according to the site of the lesion. The X-axis represents the site of the lesion, and the Y-axis represents the percentage of cases corresponding to each site. Most of the recurrent OSCC cases were of Buccal mucosa origin (74.4%), and the least common sites for recurrent OSCC were the Submandibular and submental region(5.1%) and the Gingivobuccal sulcus(5.1%) OSCC: Oral squamous cancer cells

Among the recurrent OSCC cases, Buccal mucosa (74.4%) (29) was the most commonly involved site, followed by the lateral border of the tongue(15.4%) (6). The least common sites were Submandibular and submental region(5.1%) (2) and Gingivobuccal sulcus(5.1%) (2) (Figure 3).

**Figure 4:**
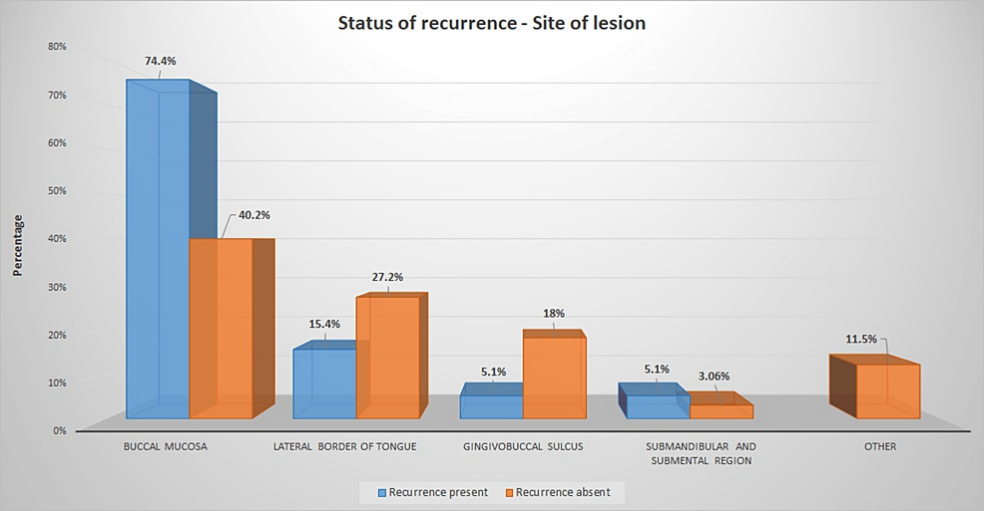
Shows the association between the status of recurrence and site of lesion among the sample population of OSCC cases. The X-axis represents the site of the lesion, and the Y-axis represents the percentage of cases. Blue represents recurrent OSCC, and orange represents the primary OSCC cases. The involvement of Buccal mucosa in recurrent OSCC cases is significantly higher than in the Primary cases, and this association was found to be statistically significant. Pearson's chi-square p-value: 0.001(p<0.05) OSCC: Oral squamous cancer cells

**Figure 5:**
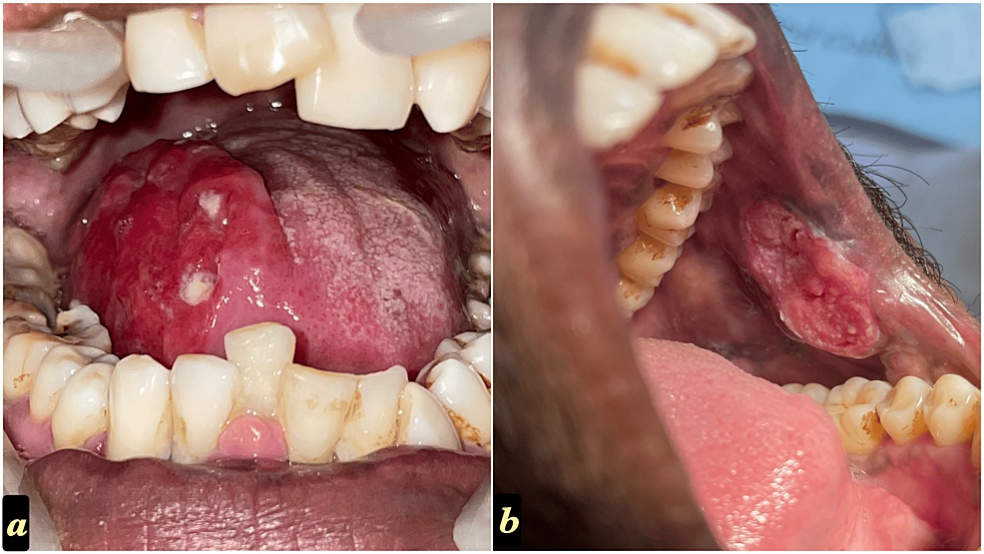
a) Primary OSCC arising on the lateral border of the tongue and b) on the left buccal mucosa near the corner of the mouth OSCC: Oral squamous cancer cells

**Figure 6:**
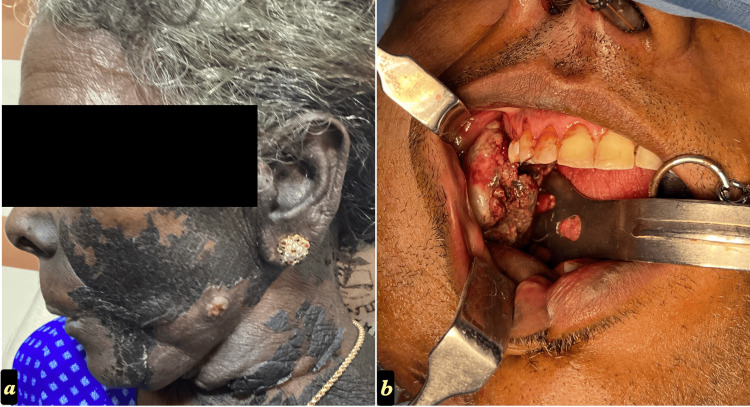
Recurrent OSCC, clinically presented as ulceroproliferative lesions on a) Left buccal mucosa (Involving skin) and b) Right buccal mucosa of two different patients OSCC: Oral squamous cancer cells

In primary OSCC cases, buccal mucosa (40.2%) (105) was the most affected site, followed by the lateral border of the tongue(27.2%) (71), gingivobuccal sulcus (18%) (47) and submandibular- submental region (3.06%) (8). Sites other than this area are represented as Other (11.5%) (30). The association analysis between the status of recurrence and the site of the lesion showed that Buccal mucosa is more commonly affected in recurrent OSCC (74.4%, 29/39) than non-recurrent OSCC (40.2% 105/261). In contrast to the results mentioned above, the lateral border of the tongue and gingivobuccal sulcus are more commonly affected in non-recurrent OSCC (27.2%, 71/261) than recurrent OSCC cases(15.4%, 6/39). This difference in site predilection in recurrent and non-recurrent cases of OSCC was statistically significant with Pearson chi-square p-value=0.001 (Table 1, 2 and Figure 4). Clinical pictures of lesional sites of primary OSCC and recurrent OSCC are shown in (Figures 5-6), respectively.

**Table 1:** Shows the cross-tabulation of cases according to the site of the lesion in recurrent OSCC and primary OSCC OSCC: Oral squamous cancer cells

		Site of the lesion					Total
		Buccal mucosa	Lateral border of tongue	Gingivobuccal sulcus	Submandibular-submental region	Others	
Status of recurrence	Recurrence present	29	6	2	2	0	39
	Recurrence absent	105	71	47	8	30	261
Total		134	77	49	10	30	300

**Table 2:** Shows Pearson hi-square value, Likelihood ratio, and Linear-by-linear association of recurrent and non-recurrent OSCC cases OSCC: Oral squamous cancer cells

Chi-Square Tests			
	Value	df	Asymptotic Significance (2-sided)
Pearson Chi-Square	19.056^a^	4	.001
Likelihood Ratio	22.981	4	.000
Linear-by-Linear Association	12.369	1	.000
N of Valid Cases	300		

## Discussion

The overall percentage of oral cancer prevalence is 2%-4% worldwide, and oral squamous cell carcinoma is reported as the sixth most common cancer. The prevalence of morbidity and mortality among males is 6.6/100000-3.1/100000, respectively, and for females, it is 2.9-100000-1.4/100000 [3,4]. The etiology of OSCC is diverse and grossly includes deleterious habits like tobacco chewing, genetic impairments, Human papillomavirus infection, and nutritional deficiencies [19]. 

The prognosis of OSCC varies based on variable parameters such as depth of invasion, the pattern of invasion, tumor budding, inflammatory reaction, and even the site of the lesion [3,20]. Conventional oral squamous cell carcinoma treatment includes surgery, chemotherapy, and radiotherapy [10]. The primary treatment option for OSCC is surgery, and the major risk factor for the recurrence of oral squamous cell carcinoma post-operatively is the failure to achieve a clear surgical margin during surgery [10,21]. The recurrence of OSCC in the postoperative phase has a poor prognosis and leads to poor quality of life for the patient [22]. 

The chiefly encompassed sites for local recurrence of OSCC are the tongue, followed by gingiva and buccal mucosa [3]. T stage and N stage were significant factors influencing regional recurrence in OSCC [23]. Clinicopathologic data from patients, such as tumor sites, clinical and pathologic stages, histological grade, invasion mechanism, and perineural invasion, have also been found to affect local or distant recurrences [3]. The site of the tumor may be critical for local control. Previous research stated that the tumors on the oral floor and buccal mucosa had worse local control than other sites within the oral cavity [18,24]. 

The current study evaluated gender predilection among the sample population and found that males are more commonly affected by OSCC. This followed previous studies [25-27]. This male predominance is due to the increased use of tobacco in males than females. However, the current scenario is changing due to the increased use of tobacco products by females [28,29]. Considering the total sample population selected, 13% (39) were recurrent OSCC. Though this seems less when compared to the non-recurrent OSCC cases (87% 261), the rate of recurrence is significant when considering the treatment modalities incurred. 

Recurrent OSCC was more commonly seen in the buccal mucosa, followed by the tongue, and least commonly seen in the gingivobuccal sulcus and submandibular and submental regions. This observation is in contrast to the previous literature, where the most common site for local recurrence of OSCC was tongue, followed by buccal mucosa [30]. These contradictory results could be due to location specificity since the geographical variations in tobacco chewing habits among patients can lead to varied clinical presentations. Buccal mucosa predilection in the present study can also be attributed to the smaller sample size in the present study. Since the population available for sampling is less, it can lead to sampling bias in the case of recurrent OSCC groups. Tobacco chewing habits and place of keeping tobacco in the oral cavity among the local population can also contribute to the site specificity for buccal mucosa in recurrent OSCC. Since the tongue is a mobile structure, lingual pain can be a considerate reason for early consultation in the case of OSCC of the tongue when compared to the buccal mucosa. This early diagnosis could be the reason for the reduced recurrence of OSCC of the tongue compared to buccal mucosa in the present study. Since we considered the site of the lesion as our primary parameter and did not include the extent of the lesion, this can act as a confounding factor in the present study. A lesion with multiple site involvement can also be as extensive as involving the entire buccal mucosa, sometimes perforating the skin rather than limited to a specific area. Segregating OSCC based on a single site alone will not solve this error, but the extent of the lesion should be considered. Hence, considering all factors, late diagnosis, anatomical variations, and extensive lesions could be reasons for the high local recurrence in buccal mucosa compared to lateral border of the tongue. Few previous studies have also analyzed the spatial patterns of recurrence of OSCC from different locations, and they found that buccal OSCC was more prone to local recurrence while tongue OSCC was prone to regional recurrence [30]. However, the biological mechanism behind this observation is still unclear and needs further study. Recently, studies have emerged evaluating the relationship between the recurrence rate and recurrence site.

Limitations of the present study are low sample size, location-specific sampling, and lack of standard specification for evaluating the site of the lesion. The population of the current study was limited to patients reported to our institution; this limits the generalisability of the results. Even though the sample size is limited, this can be considered one of the few preliminary retrospective studies conducted so far. Hence, a comparative analysis of the results of studies of the same objective will be more reliable. To overcome the limitations of small-scale studies, multiple large-scale studies, both location-specific and nonspecific, should be conducted, and the results should be compared to arrive at a precise conclusion.

## Conclusions

The present study concluded that males have a high predilection for OSCC, and the recurrent cases account for 13% (39) of the sample population (300). Even though buccal mucosa was the most common site of lesion in both recurrent and non-recurrent OSCC, the variation among both groups was statistically significant. However, the current study carries a small sample size and a location-specific sampling. Hence, further studies have to be conducted with a large sample size to test the significant correlation between the site and the rate of recurrence among patients who are diagnosed with OSCC.
